# Arbuscular Mycorrhizal Fungus Alters Alfalfa (*Medicago sativa*) Defense Enzyme Activities and Volatile Organic Compound Contents in Response to Pea Aphid (*Acyrthosiphon pisum*) Infestation

**DOI:** 10.3390/jof8121308

**Published:** 2022-12-16

**Authors:** Yajie Wang, Yingde Li, Zhen Tian, Tingyu Duan

**Affiliations:** 1 State Key Laboratory of Herbage Improvement and Grassland Agro-Ecosystems, Lanzhou Unviersity, Lanzhou 730020, China; 2 Key Laboratory of Grassland Livestock Industry Innovation, Ministry of Agriculture and Rural Affairs, Lanzhou 730020, China; 3 College of Pastoral Agriculture Science and Technology, Lanzhou University, Lanzhou 730000, China; 4 Center for Grassland Microbiome, Lanzhou University, Lanzhou 730000, China

**Keywords:** *Rhizophagus intraradices*, pea aphid, alfalfa, volatile organic compounds, RNA-seq

## Abstract

Pea aphid (*Acyrthosiphon pisum*) infestation leads to withering, reduced yield, and lower quality of the host plant. Arbuscular mycorrhizal (AM) fungi have been found to enhance their host plants’ nutrient uptake, growth, and resistance to biotic stresses, including pathogen infection and insect pest infestation. Therefore, we evaluated the effects of AM fungus *Rhizophagus intraradices* on alfalfa defense responses to pea aphid infestation. Aphid infestation did not affect the colonization of AM fungus. The inoculation of AM fungus, on average, enhanced alfalfa catalase and the contents of salicylic acid and trypsin inhibitor by 101, 9.05, and 7.89% compared with non-mycorrhizal alfalfa, respectively. In addition, polyphenol oxidase activities significantly increased by six-fold after aphid infestation in mycorrhizal alfalfa. Moreover, the fungus significantly (*p* < 0.05) improved alfalfa shoot N content, net photosynthetic and transpiration rates, and shoot dry weight in aphid infected treatment. The aphid infestation changed the total volatile organic compounds (VOCs) in alfalfa, while AM fungus enhanced the contents of methyl salicylate (MeSA). The co-expression network analysis of differentially expressed genes (DEGs) and differentially expressed VOCs analysis showed that three DEGs, namely MS.gene23894, MS.gene003889, and MS.gene012415, positively correlated with MeSA both in aphid and AM fungus groups. In conclusion, AM fungus increased alfalfa’s growth, defense enzyme activities, hormones, and VOCs content and up-regulated VOC-related genes to enhance the alfalfa’s resistance following aphid infestation.

## 1. Introduction

Aphids (*Aphidoidea*) are important insect pests that cause severe economic and agricultural damage worldwide through direct feeding and virus transmission as vectors [1]. Aphids feeding has caused an estimated production loss of 25% in alfalfa worldwide [2], of which, pea aphid (*Acyrthosiphon pisum*), cowpea aphid (*Aphis craccivora*), and spotted alfalfa aphid (*Therioaphis trifolii*) cause about 20–30% yield loss of alfalfa production in China [3]. Among them, the pea aphid feeds on legumes such as pea (*Pisum sativum*), alfalfa (*Medicago sativa*), white clover (*Trifolium repens*) and red clover (*Trifolium pratense*) [4,5,6], causing withering and death of the host plants [2].

The infestation of aphids also decreases photosynthesis [7] and photosynthates’ transport to the roots [8]. For example, feeding by *Spodoptera exigua* significantly improved net photosynthetic rate and transpiration rate of mycorrhizal plants of peanut (*Arachis hypogaea*) and tomato (*Lycopersicum esculentum*) [7]; bird cherry-oat aphid (*Rhopalosiphum padi*) infection dramatically decreased C allocation to symbionts of wheat root and arbuscular mycorrhizal (AM) fungus (*Rhizophagus irregularis*). Insect feeding usually activates a plant’s defense-related enzymes, such as superoxide dismutase (SOD), peroxidase (POD), and catalase (CAT) [9,10]. For example, the activities of CAT and SOD were significantly increased in soybeans resistant to the southern green stink bugs (*Nezara viridula*) [11]. Host plant *Medicago truncatula* responds to pea aphid infestation by adapting the jasmonic acid (JA) and salicylic acid (SA) contents [12], and aphid infestation up-regulates the expression of SA pathway-related genes, while in alfalfa, the aphid infestation increases SA concentration by 15.5% and enriches sesqui- and tri-terpenoid biosynthesis pathways [13].

Plant-produced volatile organic compounds (VOCs) are important chemical communication between plants and insects [14]. An insect pest infestation can also change the host plant’s volatile organic compounds (VOCs) [15], which repel insect herbivores or attract their natural enemies [16], hence protecting plants against biotic stress [17]. For instance, increased methyl salicylate (MeSA) in tobacco (*Nicotiana tabacum*) infected with potato aphid (*Macrosiphum euphorbiae*) caused the attraction of the aphid parasitoid *Aphidius ervi*, which impaired the development of the pest [18]. Pea aphids feeding on broad bean (*Vicia faba*) also caused the release of plant volatiles that helped the legume to resist the pests by attracting the parasitic wasp *A. ervi* [19]. An SPME-GC-MS method found that a total of 99 volatile metabolites were detected in the leaf of alfalfa [20]. It is important to clarify that alfalfa VOCs related defense to aphid infestation. In addition, the shoot tissues of alfalfa are directly used for livestock feeding; thus, pesticides are not recommended for aphid control. 

Microorganisms have potential for biocontrol of alfalfa insect pests [13]. Arbuscular mycorrhiza (AM) colonization occurs widely in vascular plants, including alfalfa (*Medicago* spp.) [21]. AM fungi colonization in plants promotes nutrient uptake, photosynthesis, and growth [22], and it enhances defense enzyme activities [23]. AM fungi can enhance plants against insects by improving plant growth, increasing plant defense enzyme activities [13], and regulating plant VOCs. For example, the mixed AM fungi colonization promotes the release of green-leaf volatiles (GLVs) in broad bean such as 3-hexenyl acetate, hexyl acetate, and MeSA [24], but it reduces the contents of sesquiterpene, (E)-caryophyllene, and (E)-β-farnesene [25]. Therefore, the changes in plants caused by the AM fungus *Rhizophagus intraradices*’ colonization affect alfalfa resistance pathogens and insect pests. In addition, the colonization of plants by AM fungus affects the mediation of the host plant’s JA, SA, and ABA contents [13]. Transcriptome analysis also shows that the AM fungus *R. intraradices* increases the expression of resistance-related genes involved in pathogenesis-related protein in alfalfa’s (*M. sativa*) response to *Phoma medicagonis* infection [26]. 

Alfalfa is one of the most important perennial forage legumes with the largest cultivation area in the world [27]. AM fungus colonization and aphid infection often occur simultaneously in alfalfa; the alfalfa–AM fungus–aphid system is common in nature. Aphid feeding causes withering, yield loss, and death of the host plants. However, the manner in which AM fungi regulates the defense mechanism of the host plant, especially alfalfa’s defense enzyme and VOCs’ related mechanism to aphid infestation, remains unclear. Therefore, the present study was designed to analyze the effects of *R. intraradices* on alfalfa’s responses to pea aphid infestation. We determined the plant growth, photosynthesis, phosphorus (P) and nitrogen (N) uptake, plant defense enzyme activities, hormone contents, and VOCs’ contents and their related key genes. We hypothesized that AM fungus enhances alfalfa’s photosynthesis and increases its defense-enzyme activities and hormone concentrations, as well as changes the VOCs’ contents and related gene expression, thus affecting alfalfa’s response to pea aphid infestation.

## 2. Materials and Methods

### 2.1. Alfalfa, AM Fungus, and Growth Medium

Seeds of the alfalfa (*Medicago sativa*) cultivar “Longdong” were obtained from the Forage Seed Testing Centre, Lanzhou, Ministry of Agriculture, and Rural Ministry of China, while the AM fungus (*Rhizophagus intraradices*) was purchased from the Bank of Glomeromycota in Beijing, China. The inoculum consisted of dry soil containing AM fungus spores (>100 spores per g), mycelium, and white clover root fragments, which were prepared in pot cultures of AM fungus grown on the white clover (*Trifolium repens*) in the greenhouse. The alfalfa growth medium was mixed with pre-sieved sand and soil using a 2 mm sieve at a 1:3 weight ratio. Sand and soil were purchased from the flower market in Lanzhou, China. All the growth medium components were sterilized by autoclaving at 121 °C for 1 h twice in three days [26].

### 2.2. AM Fungus Inoculation and Aphid Infestation

A two-stage experiment was established using alfalfa and aphids in a plant incubator (RXM-508C; Ningbo Jiangnan Instrument Factory, Ningbo, China) with 12 fluorescent lights (300 μmol·m^−2^·s^−1^) at 25 °C and 12 h of dark at 25 °C (watered with tap water every three days). The alfalfa seeds were surface-sterilized with 10% hydrogen peroxide (H_2_O_2_) for 10 min and then rinsed three times with sterile distilled water before germinating on wet filter paper in the dark at 25 °C for 48 h. Before transplantation, 50 g of AM fungus were added to the growth medium at a depth of 2–3 cm in each of eight pots (AM treatment). The control pots contained the same growth medium with a 50 g sterile growth medium each (non-mycorrhizal treatment, NM). Five germinated seeds were then transplanted in a 12 × 10 × 8 cm pot and thinned to four seedlings after one week.

We captured pea aphids from an alfalfa field at the Yuzhong campus of Lanzhou University, China and then selected and reared the single adults on broad bean plants. We then identified the newly emerged aphid offspring morphologically and molecularly, as described previously [28]. At 51 days after planting (DAP), half of the alfalfa plants inoculated with or without AM fungus were infested with five similar size adult pea aphids (A+). The remaining half of the alfalfa plants were not infested with pea aphids (A−).

### 2.3. AM Colonization, Shoot N, P Uptake, Dry Weight, Defense Enzyme Activity, and Hormone Content

At harvest (60 DAP), 0.5 g of fresh shoots from each pot were used to determine the enzyme activities, by measuring polyphenol oxidase (PPO), superoxide dismutase (SOD), peroxidase (POD), and catalase (CAT), as described previously [29]. We measured the concentrations of jasmonic acid (JA), salicylic acid (SA), and abscisic acid (ABA) contents; nitric oxide (NO); total phenols; and trypsin inhibitors from 0.4 g of fresh shoots using ELISA kits (Plant JA, Plant SA, Plant ABA, Plant NO, Plant total phenols, and Plant trypsin inhibitor) following the manufacturer’s instructions (mlbio; Shanghai Enzyme-linked Biotechnology, Shanghai, China) [26]. About 1.0 g of fresh shoots from each pot was used for the VOCs’ content analysis using static headspace SPME-gas chromatography coupled with mass spectrometry (GC-MS) [30,31], while the remaining fresh shoot tissues were used to measure the dry weight. Thus, the total plant shoot dry weight was calculated from the fresh and dry weight ratios. In addition, 0.2 g of plant dry shoots were digested in 5 mL H_2_SO_4_ to determine the total N and P by a flow injection analyzer (FIAstar 5000 Analyzer; FOSS, Munkedal, Sweden) with the Kjeldahl method [32].

Approximately 0.15 g of the root tissues from each pot were used for AM colonization tests as previously reported [33]. Roots were cut into 1 cm-long pieces and cleaned in 10% KOH for 40 min at 60 °C water bath. The samples were then treated in 1 M HCl for 30 s, washed three times with distilled water, and stained in trypan blue overnight at room temperature. They were then washed three more times with distilled water and maintained in a 1:1:1 solution of 5% lactic acid: glycerin: water (*v*/*v*/*v*). The root samples were then used to determine the degree of mycorrhizal colonization using a compound microscope (SOFTOP BH200M-R, Ningbo, China) [33,34].

### 2.4. The Photosynthesis Indexes

The net photosynthetic and transpiration rates were measured at 50 DAP (before aphid infesting) and 59 DAP (after aphid infesting) using a portable photosynthesis system (LI-6400, LI-COR, Inc., Lincoln, NE, USA). The readings were taken between 9:00 and 11:00 am with 1200 μmol·m^−2^·s^−1^ of PAR saturation [35].

### 2.5. The Transcriptome Analysis for Plant Leaves

At harvest (60 DAP), 0.1 g of fresh shoots from the four seedlings in each pot was randomly collected, flash-frozen immediately in liquid nitrogen, and stored at −80 °C before total RNA extraction. The total RNA was isolated from the leaf tissues of three biological replicates per treatment (NMA−, NMA+, AMA−, AMA+) using TRIzol Reagent following the manufacturer’s instructions (Thermo Fisher Scientific, Waltham, MA, USA). The residual genomic DNA was removed from the RNA samples using DNase I (Takara Bio, Kusatsu-Shiga, Japan). RNA quality and quantity were determined using the 2100 Bioanalyser (Agilent Technologies, Santa Clara, CA, USA) and ND-2000 (NanoDrop Technologies, Wilmington, DE, USA). The high-quality RNA sample (OD_260/280_ = 1.8–2.2, OD_260/230_ ≥ 2.0, RIN ≥ 6.5, 28S:18S ≥ 1.0, >2 μg) was selected to construct the sequencing library [36].

Accordingly, 1 μg of the total RNA was used to construct the RNA-seq transcriptome libraries using the TruSeq^TM^ RNA sample preparation kit following the manufacturer’s protocol (Illumina, San Diego, CA, USA). The mRNA was isolated by the polyA selection method using oligo(dT) beads and fragmented using the fragmentation buffer. The double-stranded cDNA was synthesized using random hexamer primers (Illumina, San Diego, CA, USA), following the protocol of the SuperScript double-strand cDNA synthesis kit (Invitrogen, Waltham, MA, USA). The synthesized cDNA was end-repaired and phosphorylated, and the ‘A’ base was enriched following the Illumina library construction protocol. After cDNA quantification, 200–300 bp target fragments were amplified by PCR using Phusion DNA polymerase (New England Biolabs, Ipswich, MA, USA) for 15 PCR SeqPrep cycles. The libraries were paired-end sequenced on the Illumina HiSeq xten/NovaSeq 6000 sequencer (2 × 150 bp read length) [37]. 

The raw paired-end reads were trimmed by SeqPrep (https://github.com/jstjohn/SeqPrep, accessed on 9 October 2022) and quality controlled by Sickle (https://github.com/najoshi/sickle, accessed on 9 October 2022) using default parameters. The clean reads were mapped to the *M. sativa* reference genome (https://figshare.com/projects/whole_genome_sequencing_and_assembly_of_Medicago_sativa/66380, accessed on 9 October 2022) using the TopHat (http://tophat.cbcb.umd.edu/, version 2.0.0, accessed on 9 October 2022) software [38]. The generated raw sequence dataset was submitted to the National Center for Biotechnology Information (NCBI) in the Short Read Archive (SRA) database under accession number PRJNA832072. 

RSEM (http://deweylab.biostat.wisc.edu/rsem/, accessed on 9 October 2022) was used to normalize the level of gene expression in fragments per kilobase of exon per million mapped reads (FPKM). Analyses of the differentially expressed genes (DEGs) between two different samples (NMA− vs. AMA−, NMA− vs. NMA+, AMA− vs. AMA+, and NMA+ vs. AMA+) were performed using DESeq2 in the R statistical environment (*p*_adjust_ < 0.05).

### 2.6. Volatile Organic Components Analysis

For each pot, 1 g of fresh shoots was immediately frozen in liquid nitrogen after harvesting and stored at −80 °C. A total of four treatments (NMA−, AMA−, NMA+, AMA+) with four biological replicate samples were separately ground to powder in liquid nitrogen to isolate the volatiles [39]. The powder was transferred immediately to a 20 mL headspace vial containing 2 mL saturated NaCl solution (Agilent, Palo Alto, CA, USA). The vials were sealed using crimp-top caps with TFE-silicone headspace septa (Agilent). At the time of SPME analysis, each vial was placed at 60 °C for 10 min, and then a 65 µm divinylbenzene/carboxen/polydimethylsilioxan fiber (Supelco, Bellefonte, PA, USA) was exposed to the headspace of the sample for 20 min at 60 °C.

The desorption of the VOCs from the fiber coating was carried out in the injection port of the GC apparatus (Model 7890B; Agilent) at 250 °C for 5 min under the splitless mode. The VOCs were identified and quantified using an Agilent Model 7890B GC and a 7000D mass spectrometer (Agilent) equipped with a 30 m × 0.25 mm × 1.0 μm DB-5MS (5% phenyl-polymethylsiloxane) capillary column. Helium was used as the carrier gas at a linear velocity of 1.0 mL/min. The injector temperature was kept at 250 °C and the detector at 280 °C. The oven temperature was programmed from 40 °C (1 min), increasing at 5 °C/min to 280 °C. The mass spectra were recorded in electron impact (EI) ionization mode at 70 eV. The quadrupole mass detector, ion source, and transfer line temperatures were set at 150, 230, and 280 °C, respectively. The mass spectra were scanned in the range of *m*/*z* 30–350 amu at 1 s intervals. The volatile compounds were identified by comparing the mass spectra with the data system library (NIST) and linear retention index [40].

Principal component analysis (PCA) was used to differentiate the samples and identify marker metabolites. Afterwards, the variable influence on projection (VIP) was used to illustrate the variables that contributed to the separation. The orthogonal partial least squares discriminant analysis (OPLS-DA) extension was then used to compute the variation of X, which is predictive (correlation between X and Y), and the part of the variation which is orthogonal (unrelated) to Y [41].

### 2.7. Statistical Analysis 

Data of AM colonization; fresh weight; dry weight; total N; total P; JA; SA; NO; ABA; phenolic and trypsin inhibitor concentrations; and the activities of CAT, POD, SOD, and PPO are presented as the means ± standard error of the mean (SEM) of four biological replicates. Data of the net photosynthetic and transpiration rates are presented as SEM of 4 biological replicates. The data were subjected to analysis of variance (ANOVA) using the R statistical program (version 4.2.0). Comparisons between the means were determined by Turkey’s HSD test (*p* < 0.05). The percentage of AM colonization and net photosynthetic rate were SQRT transformed while the transpiration rate was log transformed to achieve normality. The RNA-seq analysis was performed using the free online platform of Majorbio Cloud Platform (www.majorbio.com, accessed on 9 October 2022). The VOCs analysis was performed using the free online platform of Metware Cloud (cloud.metware.cn, accessed on 9 October 2022).

## 3. Results 

### 3.1. AM Fungal Colonization, Plant Growth, Shoot N and P Uptake

**Figure 1:**
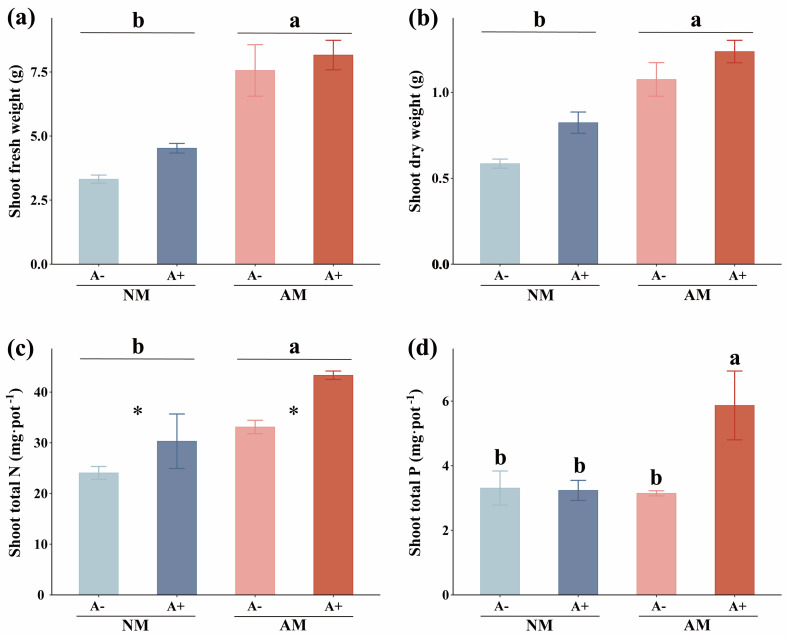
(**a**) Shoot fresh weight, (**b**) Shoot dry weight, (**c**) Shoot total N, and (**d**) Shoot total P of *M. sativa* inoculated with *R. intraradices* (AM) and infested by *A. pisum* (A+) or without inoculation with *R. intraradices* (NM) and non-infested with *A. pisum* (A−). All values are shown as means ± SEM of four biological replicates. Different letters above bars (**d**) or pairs of bars (**a**–**c**) represent significant difference in the comparison at *p* < 0.05, asterisks indicate significant differences between A− and A−, as determined by a Tukey’s HSD test. SEM, standard error of the mean.

**Figure 2:**
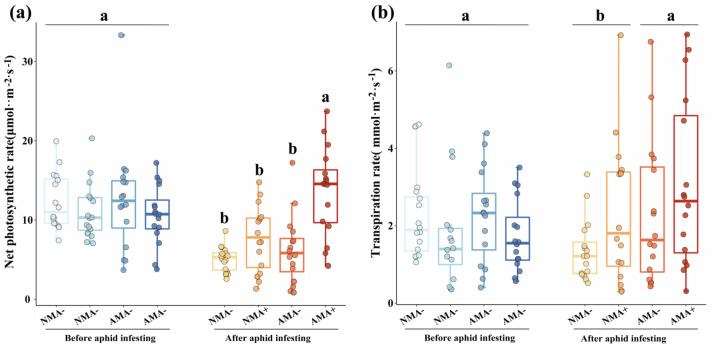
(**a**) Net photosynthetic rate and (**b**) transpiration rate of *M. sativa* inoculated with *R. intraradices* (AM) and infested by *A. pisum* (A+) or without inoculation with *R. intraradices* (NM) and un-infested by *A. pisum* (A−) before aphid infesting and after aphid infesting. Boxes show first quartile, median, and third quartile. Whiskers extend to the most extreme points within 1.5 × box lengths, and the points are values that fall outside the whiskers. Different letters above bars or pairs of bars represent significant difference between different treatments before aphid infesting or after aphid infesting in the comparison at *p* < 0.05, as determined by a Tukey’s HSD test.

The mean percentags of AM fungus colonization were 45% and 43.33% in the mycorrhiza-inoculated alfalfa without and with pea aphid infestation, respectively. No mycorrhizal structures were observed in the non-fungal-inoculated plant roots. The infestation of aphids did not significantly affect AM colonization (Figure S1; *p* > 0.05). AM fungus increased alfalfa shoot fresh weight by 100% (Figure 1a; *p* = 0.0043) and shoot dry weight by 64.15% (*p* = 0.0151; Figure 1b). Moreover, the alfalfa shoot total N was increased 40.60% by AM fungus (*p* = 0.0083), and mycorrhizal alfalfa with aphid infestation had the highest shoot total N (Figure 1c) and shoot total P (*p* = 0.0373; Figure 1d). AM fungus significantly increased the net photosynthetic rate of alfalfa infested by aphids (*p* < 0.0001; Figure 2a), and the transpiration rate of mycorrhizal alfalfa at 45.00% was higher than that of non-mycorrhizal alfalfa at 59 DAP (*p* = 0.03867; Figure 2b) (Table S1), which is consistent with the higher biomass of mycorrhizal plants.

### 3.2. The Activities of Plant Defense Enzymes, Jasmonic Acid, SA, Trypsin Inhibitors, Total Phenolics, ABA, and NO Concentration in Alfalfa

**Figure 3:**
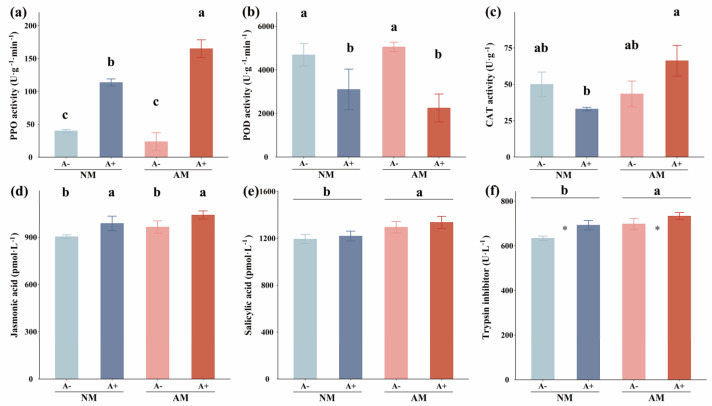
(**a**) PPO activity, (**b**) POD activity, (**c**) CAT activity, (**d**) JA concentration, (**e**) SA concentration, and (**f**) Trypsin inhibitor concentration of *M. sativa* inoculated with *R. intraradices* (AM) and infested by *A. pisum* (A+) or without inoculation with *R. intraradices* (NM) and non-infested with *A. pisum* (A−). All values are shown as means ± SEM of four biological replicates. Different letters above bars (**a**–**d**) or pairs of bars (**e**,**f**) represent significant difference in the comparison at *p* < 0.05, asterisks indicate significant differences between A− and A−, as determined by a Tukey’s HSD test. SEM, standard error of the mean. PPO, polyphenol oxidase; POD, peroxidase; CAT, catalase; JA, jasmonic acid; SA, salicylic acid.

AM fungus alone did not affect plant defense enzyme activity; however, its interaction with aphid-affected plant CAT and PPO activities indicates that AM fungus plays an important role only when the plant is exposed to aphid stress (Table S1). Aphid infestation significantly increased plant CAT and PPO, especially in mycorrhizal plants. The PPO activities were significantly increased by 184% and 600% after aphid infestation in non-mycorrhizal alfalfa and mycorrhizal alfalfa, respectively (*p* = 0.0054) (Figure 3a). The CAT activity in mycorrhizal alfalfa was 101% higher than in non-mycorrhizal plants after aphid infestation (*p* = 0.0304; Figure 3c). However, pea aphid infestation decreased the POD activity (*p* = 0.0046; Figure 3b) but did not affect the SOD activity in alfalfa plants (Figure S2) (Table S1). Aphid infestation significantly increased JA concentration by 8.59% (*p =* 0.0335; Figure 3d), and AM fungus colonization increased SA concentration by 9.05% in alfalfa (*p* = 0.0337; Figure 3e). There was no interactive effect of aphid infestation and AM colonization on JA and SA. Trypsin inhibitor concentration in mycorrhizal alfalfa was 7.89% higher in non-mycorrhizal plants (*p* = 0.0174). Furthermore, aphid infestation significantly increased trypsin inhibitor concentration in non-mycorrhizal alfalfa, while AM fungus increased trypsin inhibitors either in aphid-infested or non-infested alfalfa (Figure 3f; Table S1). This indicates that AM fungus colonization activated the plant’s trypsin-inhibitor-related defense process. However, there were no significant differences in the contents of total phenols, ABA, and NO across the treatments (Figure S3).

### 3.3. Volatile Organic Components in Alfalfa

**Figure 4:**
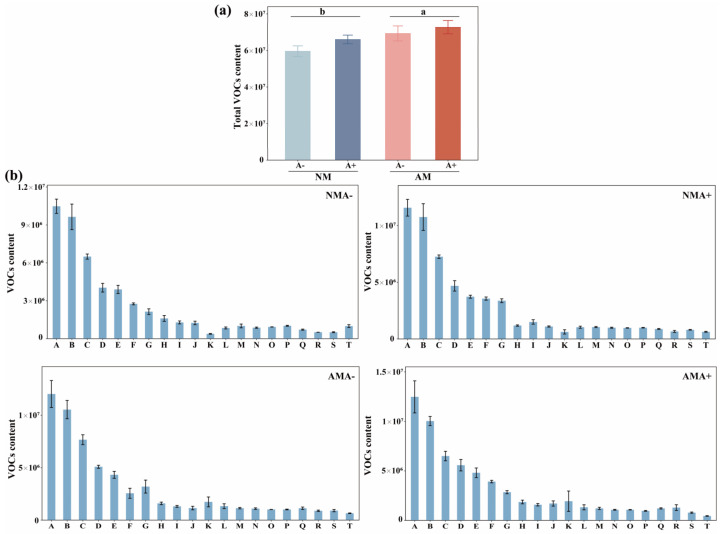
(**a**) Total VOC content from *M. sativa* inoculated with *R. intraradices* (AM) and infested by *A. pisum* (A+) or without inoculation with *R. intraradices* (NM) and non-infested with *A. pisum* (A−). All values are shown as means ± SEM of four biological replicates. Different letters above pairs of bars represent significant difference in the comparison at *p* < 0.05 (**b**) Individual VOCs’ contents in NMA−, NMA+, AMA−, AMA+ treatments. Means ± SEM for the 20 most abundant compounds. The same compounds are present in each treatment, and the relative contents of compounds are similar. A, 2-Hexenal, (E)−; B, 2-Ethyl-1-hexanol; C, 3-Buten-2-one, 4-(2,6,6-trimethyl-1-cyclohexen-1-yl)-; D, 2,4-Heptadienal, (E,E)-; E, 1-Hexanol; F, Hexanal; G, 3-Buten-2-one, 4-(2,2,6-trimethyl-7-oxabicyclo[4.1.0]hept-1-yl)-; H, 3,5-Octadien-2-one; I, 1-Octen-3-ol; J, Phenylethyl Alcohol; K, 2-Penten-1-ol, (Z)−; L, Benzyl alcohol; M, 1-Cyclohexene-1-carboxaldehyde, 2,6,6-trimethyl-; N, Cyclohexanol; O, 4-Heptanone, 2,6-dimethyl-; P, Pentadecane; Q, Benzaldehyde; R, 2,6-Nonadienal, (E,Z)−; S, Cyclohexanol, 2,6-dimethyl-; T, Octanoic acid, ethyl ester.

One hundred fourteen VOCs were identified, including terpenes, alcohol, aromatics, heterocyclic compounds, aldehyde, ketone, alkanes, ester, phenol, ether, and olefin. The differential VOCs were satisfied by a VIP > 1 and a *p* value < 0.05 in different samples. The colonization of AM fungus increased the total contents of VOCs (*p* = 0.0296; Figure 4a) in alfalfa plants. The top 20 VOCs with the highest mean content in the four treatments were 2-Hexenal, (E)−; 2-Ethyl-1-hexanol; 3-Buten-2-one; 4-(2,6,6-trimethyl-1-cyclohexen-1-yl)-; 2,4-Heptadienal, (E,E)−; 1-Hexanol; Hexanal; 3-Buten-2-one, 4-(2,2,6-trimethyl-7-oxabicyclo [4.1.0]hept-1-yl)-; Cyclohexanol; 3,5-Octadien-2-one; 1-Octen-3-ol; Phenylethyl Alcohol; 2-Penten-1-ol, (Z)-; Benzyl alcohol; 1-Cyclohexene-1-carboxaldehyde, 2,6,6-trimethyl-; 4-Heptanone, 2,6-dimethyl-; Pentadecane; Benzaldehyde; 2,6-Nonadienal, (E,Z)−; Cyclohexanol, 2,6-dimethyl-; and Octanoic acid, ethyl ester (Figure 4b).

Seven different VOCs, including three esters, two alcohols, one aldehyde, and one alkane, were detected in four groups (NMA− vs. AMA−, NMA− vs. NMA+, AMA− vs. AMA+ and NMA+ vs. AMA+). The amounts of butanedioic acid, methyl ester, and dimethyl ester were higher in mycorrhizal alfalfa than in non-mycorrhizal alfalfa plants. Pea aphid infestation decreased 2-Octen-1-ol, (E)− and MeSA in the non-mycorrhizal alfalfa leaves but increased the concentration of Pentadecanal, (E)-2,6-dimethylocta-3,7-diene-2,6-diol; Nonane, 5-(2-methylpropyl)-; and 3-Hexen-1-ol, acetate, (E)− in the plants inoculated with mycorrhiza and infested with pea aphids. The concentrations of 2-Octen-1-ol, (E)−; MeSA; (E)-2,6-Dimethylocta-3,7-diene-2,6-diol; and nonane, 5-(2-methylpropyl)- were higher in the mycorrhizal alfalfa infested with pea aphids than the non-mycorrhizal alfalfa infested with aphids (Table 1).

The PCA showed AM fungus colonization, and pea aphid infestation affected VOC composition and content. The VOCs were highest in the pea aphids fed on the mycorrhizal alfalfa. A part of VOCs was consistent in four different treatments (Figure S4). The OPLS-DA showed the AMA− and AMA+ treatments were separated from the other treatments, while the NMA- and AMA+ treatments were separated from the NMA+ and AMA− treatments (Figure S5a). Sixty compounds had a high discriminatory power (i.e., VIP > 1), and all were associated with the four treatments, the top 20 listed by decreasing order of VIP value, with numbers referring to the volatile compounds listed in Table S2: (**76**), (**44**), (**109**), (**49**), (**39**), (**59**), (**114**), (**12**), (**88**), (**68**), (**72**), (**50**), (**40**), (**1**), (**79**), (**17**), (**55**), (**48**), (**112**) and (**28**) (Figure S5b).

### 3.4. Differentially Expressed Genes and VOCs Analysis

**Figure 5:**
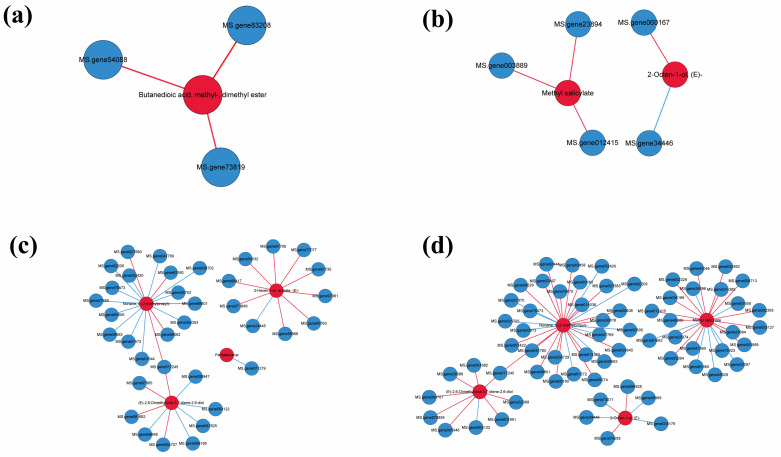
Co-expression network and expression profiles for each module of differentially expressed genes and differentially expressed VOCs in (**a**) NMA− vs. AMA−, (**b**) NMA− vs. NMA+, (**c**) AMA- vs. AMA+, (**d**) NMA+ vs. AMA+. Nodes colored in ‘red’ and ‘blue’ represent DEMs and differentially expressed VOCs. Edges colored in ‘red’ and ‘blue’ represent positive and negative correlations. VOCs, volatile organic components.

The co-expression network analysis of differentially expressed genes (DEGs) and differentially expressed VOCs was conducted to examine the relationship between DEGs and differentially expressed VOCs in the aphid-infested alfalfa plants (Pearson correlation coefficient > 0.8 or < −0.8). Sixty-six genes were found to be associated with four metabolites in NMA+ vs. AMA+, of which MS.gene071245 positively regulated (E)-2,6-Dimethylocta-3,7-diene-2,6-diol and nonane, 5-(2-methylpropyl)- in NMA+ vs. AMA+. Similarly, three DEGs were found to be associated with butanedioic acid, methyl ester, and dimethyl ester in NMA− vs. AMA−. Moreover, three and two DEGs were associated with 2-Octen-1-ol, (E)− and MeSA in NMA- vs. NMA+; MS.gene060167 positively regulated 2-Octen-1-ol, (E)−, while MS.gene34446 negatively regulated 2-Octen-1-ol, (E)−. In contrast, only MS.gene071279 was found to be associated with pentadecanal while MS.gene071245 positively regulated (E)-2,6-Dimethylocta-3,7-diene-2,6-diol and nonane, 5-(2-methylpropyl)- in AMA− vs. AMA+ (Figure 5).

A total of three and 22 DEGs were associated with MeSA in NMA− vs. NMA+ and NMA+ vs. AMA+, respectively. In total, there are three DEGs that positively correlated with MeSA (MS.gene23894, MS.gene003889, and MS.gene012415) both in NMA− vs. NMA+ and NMA+ vs. AMA+. These three DEGs associated with MeSA in NMA− vs. NMA+ were down-regulated, while these DEGs in NMA+ vs. AMA+ were up-regulated. Moreover, the rest of the 12 DEGs positively correlated with MeSA up-regulated in NMA+ vs. AMA+, and seven DEGs negatively correlated with MeSA down-regulated in NMA+ vs. AMA+, respectively (Tables S3 and S4). These results were consistent with the content of MeSA in different treatments.

## 4. Discussion

The present study reveals that the biochemical mechanism of AM fungus modulates plant responses to pea aphid infestation. We did not observe the negative influence of aphids on the growth of non-mycorrhizal and mycorrhizal plants. We assume this may be due to the short period of the experiment. In addition, alfalfa plants may have a compensation for adverse effects of aphid infestation. The infestation of the aphids induced alfalfa to absorb P and N in mycorrhiza-inoculated plants, which may compensate for the loss caused by aphids feeding on plant growth as revealed by higher fresh and dry weights in AMA+. Furthermore, the increased shoot total P content in plant leaves can increase the net photosynthetic rate in plants [42]. This is consistent with the higher net photosynthesis in mycorrhiza-inoculated plants infested with pea aphids. Insect feeding usually decreases plant photosynthesis, reducing the transportation of photosynthesis to the host plant’s roots [8]. Subsequently, this affects the colonization of the AM fungi and nutrient acquisition from the host plants [7]. Alfalfa was exposed to pea aphid stress as the symbiotic relationship changed owing to the carbon–lipid exchange with N and P absorption by the AM fungus [43]. Our results show AM colonization was not affected by aphid infestation, which shows the symbiosis of AM fungus and alfalfa was stable, and mycorrhiza retains its function in enhancing plant P uptake as this was proved by the higher P and N content in mycorrhizal plants. Babikova et al. found that the alteration of metabolism of alfalfa by the AM fungus antagonized the infestation of pea aphids by accumulating total P in the leaves [44]. In our study, the stable AM fungal colonization in alfalfa protected plants from aphid infestation by nutrient uptake and higher photosynthetic rate.

Our results show aphid infestation causes alfalfa defense enzyme activity, especially reactive oxygen species (ROS) related enzymes, such as PPO, POD, and CAT; this agrees with previous studies [45,46,47,48]. For example, Mahanil found the ROS related enzyme was increased when tomato was exposed to the common cutworm [46]. Another study found host plant POD activity increases to resist insect infestation with identification of B6T173 (ZmPrx35) as the prevailing peroxidase in highly insect-resistant maize (*Zea mays*, p84C3) kernels by activity-directed purification, which include activities of antioxidant enzymes in three species of Ludwigia weeds on feeding by *Altica cyanea*. Though ROS are produced in the plant to resist biotic stresses, a large quantity of ROS might damage nucleic acids and proteins in the cells, polyunsaturated fatty acids on the cell membranes, and subcellular organelle membranes [48]. The present study found the POD activity in alfalfa significantly decreased after pea aphid infestation, indicating reduced ROS in the legumes, which may benefit plants’ stress tolerance [49]. In addition, the activity of CAT increased in mycorrhiza-inoculated alfalfa plants infested with pea aphids compared to non-fungal-inoculated plants, which may be related to a signal for the induction of defense genes in plants [50]. Therefore, this study suggests that AM fungus could alter a plant’s response to aphids by increasing plant P, N uptake, plant growth, and defense enzyme activity.

Our research shows that JA significantly increases in the alfalfa after being infested with pea aphids, while AM fungus enhances SA concentration in the legumes. However, JA and SA did not consistently change in alfalfa in response to pea aphid infestation, with JA acting as the altering hormone in the legumes’ response to pea aphid infestation. The AM fungus-induced SA might serve as a growth, antioxidant, and metabolic activity regulator for alfalfa. Plant hormones such as JA and SA are essential endogenous molecules involved in plant development and direct and indirect defense systems [51]. Jasmonic acid and SA are the primary signaling pathways in plant defense [52,53]. The JA signaling pathway plays an essential role in plant growth, activation of plant resistance genes, and enhanced antioxidant enzyme activity [54,55]. The SA signaling pathway regulates growth, enhances the plant antioxidant system, and increases metabolic activity [56,57]. Alfalfa shows a stronger response to aphid infestation than to AM colonization, which indicates JA and SA pathways are closely related to plant response to biotic stress [13], while AM fungus mostly enhances a plant’s JA content when a plant is exposed to stress conditions. The plant defense system shares a complex relationship with the hormonal pathway, including coordination, prioritization, and cross-talk [58,59]. Trypsin inhibitors contribute to the tolerance of host plants to heavy herbivore pressure by deterring insect herbivory [60]. The high levels of trypsin inhibitors in mycorrhiza-inoculated plants and pea aphid treatments indicate trypsin inhibitors may play important roles in plant response to AM colonization and aphid infestation [13]. AM fungus could induce trypsin-inhibitor accumulation in the same way that occurs in the presence or absence of aphids. The higher rate of trypsin inhibitors in mycorrhizal plants is perhaps present before the arrival of the aphids and therefore limits the infestation of the plants by the aphids. The number of VOCs detected in this study (114) was larger than commonly reported in similar studies. For example, 17 VOCs were reported in a study of *V. faba* response to aphid attack [61], 10 in a study of *Z. mays* response to application of herbivore beetle extract [62], and 33 in a study of site differences in VOC profiles of *Calluna vulgaris* [63]. The reason for the larger number of VOCs detected here is unclear. One possibility is that SPME extraction of VOCs from frozen leaf material was more efficient than vapor-phase VOC capture in live-plant headspaces used in many studies [20].

The AM fungus induced elevated levels of overall contents and resulted in apparent changes in the ratios of seven individual volatile compounds. Many previous studies have shown that insect herbivores can exploit VOC cues to avoid inferior hosts [64,65]. Thus, despite the large sizes of mycorrhiza-inoculated alfalfa plants, more quantities of VOCs in the fungal-inoculated plants may repel aphids. Subtle quantitative and qualitative differences also exist between the VOCs’ contents in inoculated and non-mycorrhiza-inoculated alfalfa after pea aphid infestation [66]. There are ester, ketone, and terpenes in the top 20 VOCs that had a high discriminatory power in the four treatments. Among these, 3-Octanone and gamma-terpinene in alfalfa can enhance plant resistance to aphids. The 3-Octanone also elicits higher Electroantennogram (EAG) responses in the vetch aphid *Megoura viciae Buckton* (Homoptera, Aphididae) [67], while gamma-terpinene repels the insect through its toxic property [68,69].

The VOCs such as MeSA are the key compounds in mycorrhiza–alfalfa response to pea aphid infestation. MeSA is synthesized from SA mainly through carboxyl methyltransferase in the plant [70] as a long-distance signal associated with systemic acquired resistance (SAR) [71]. MeSA can induce plant growth and development and enhance defense responses [72]. Moreover, plants emit MeSA into the atmosphere [73] to repel aphids and attract their parasitoids [74,75]. MeSA attracts aphid predators, such as syrphid flies (Diptera: Syrphidae) and green lacewings (Neuroptera: Chrysopidae) to decrease Aphis glycines Matsumura (Hemiptera: Aphididae) abundance [76]. In the current study, pea aphid infestation reduced MeSA content, benefiting the insects’ infestation but reducing the predators’ discovery. AM fungus increased the total content of VOCs, which likely influenced the plants’ response to pea aphid infestation and predator attraction. The key DEGs, including MS.gene23894, MS.gene003889, and MS.gene012415, which regulate MeSA content, were down-regulated in the NMA− vs. NMA+ and up-regulated in the NMA+ vs. AMA+. Generally, MeSA increases the abundance of natural enemies in numerous food crops and has been commercially used as slow-release lures for pest control [77].

## 5. Conclusions

Colonization of the AM fungus *R. intraradices* promotes plant growth, N uptake, net photosynthesis, and transpiration rates when alfalfa is exposed to aphid infestation, thus compensating for the negative influence caused by pea aphids. The infestation of pea aphids activates alfalfa PPO activities and JA concentration, while AM fungus colonization enhances plant-defense enzyme activities when plants are exposed to pea aphid infestation, and the content of SA in mycorrhizal alfalfa is higher than non-mycorrhizal alfalfa. AM fungus changes alfalfa’s response to pea aphids, and plants have complex defense pathways in response to insect infestation. Moreover, AM fungus increases MeSA, which is decreased by pea aphid infestation. In addition, the MeSA-regulated key genes in alfalfa are up-regulated by AM fungus after pea aphid infestation.

## Figures and Tables

**Table 1:** Differentially expressed VOCs information of different compared groups.

Control	Treatment	Compounds	VIP	*p*_Value	Fold Change	Log2FC
NMA−	AMA-	Butanedioic acid, methyl-, dimethyl ester	1.596	0.00034495	4.849	2.278
NMA−	NMA+	2-Octen-1-ol, (E)−	1.280	0.026913339	0.424	1.237
NMA−	NMA+	Methyl salicylate	1.694	0.00390791	0.360	1.475
AMA−	AMA+	Pentadecanal	1.618	0.048801556	2.064	1.045
AMA−	AMA+	(E)-2,6-Dimethylocta-3,7-diene-2,6-diol	1.962	0.029184939	3.582	1.841
AMA−	AMA+	Nonane, 5-(2-methylpropyl)-	2.166	0.000394176	3.454	1.788
AMA−	AMA+	3-Hexen-1-ol, acetate, (E)−	1.866	0.045902066	3.711	1.892
NMA+	AMA+	2-Octen-1-ol, (E)−	1.433	0.031982472	3.122	1.642
NMA+	AMA+	Methyl salicylate	1.610	0.007071354	5.098	2.350
NMA+	AMA+	(E)-2,6-Dimethylocta-3,7-diene-2,6-diol	1.478	0.034961913	3.214	1.685
NMA+	AMA+	Nonane, 5-(2-methylpropyl)-	1.666	0.00029612	3.207	1.681

## Data Availability

All applicable data are published and referenced in the paper.

## Supplementary Materials

The following supporting information can be downloaded at: https://www.mdpi.com/article/10.3390/jof8121308/s1, Figure S1: AM fungi colonization of *M. sativa* inoculated with *R. intraradices* (AM) and infested by *A. pisum* (A+) or without inoculation with *R. intraradices* (NM), and un-infested by *A. pisum* (A−). All values are shown as means ± SEM of four biological replicates. Different letters above bars represent significant difference in the comparison at *p* < 0.05, as determined by Tukey’s HSD test. SEM, standard error of the mean.; Figure S2: SOD activcity of *M. sativa* inoculated with *R. intraradices* (AM) and infested by *A. pisum* (A+) or without inoculation with *R. intraradices* (NM) and non-infested with *A. pisum* (A−). All values are shown as means ± SEM of four biological replicates. Different letters above pairs of bars represent significant difference in the comparison at *p* < 0.05, as determined by a Tukey’s HSD test. SEM, standard error of the mean. SOD, superoxide dismutase.; Figure S3: (**a**) Total phenols concentration, (**b**) ABA concentration, and (**c**) NO concentration of *M. sativa* inoculated with *R. intraradices* (AM) and infested by *A. pisum* (A+) or without inoculation with *R. intraradices* (NM) and non-infested with *A. pisum* (A−). All values are shown as means ± SEM of four biological replicates. Different letters above pairs of bars represent significant difference in the comparison at *p* < 0.05, as determined by a Tukey’s HSD test. SEM, standard error of the mean. ABA, abscisic acid.; Figure S4: Principal component analysis (PCA) in the NMA−, NMA+, AMA− and AMA+ groups, respectively. The x-axis shows the first principal component. The y-axis shows the second principal component.; Figure S5: (**a**) OPLS-DA comparison of the VOCs contents in the NMA−, NMA+, AMA− and AMA+ groups. Score plot of the samples, with the percentage of explained variation in parentheses. The OPLS-DA resulted in a model with two predictive components. (**b**) Loading plot of the two components of the OPLS-DA, showing the contribution of each of the compounds towards the model. Numbers refer to the volatile compounds listed in Table S2.; Table S1: Main effects of AM fungus (inoculation or uninculation), pea aphid (infested or uninfested) and their two way interactions on AM colonization, Shoot fresh weight, Shoot dry weight, Shoot total N, Shoot total P, PPO, CAT, POD, SOD, SA, JA, ABA, NO, Trypsin inhibitor, Total phenols, Total VOCs. *p* < 0.05 are highlighted in bold.; Table S2: The compounds of 114 VOCs in *Medicago sativa* roots inoculated with Rhizophagus intraradices (AM) and infested with *Acyrthosiphon pisum* (A+) or non-inoculated with R. intraradices (NM) and non-infested with *A. pisum* (A−).; Table S3: Methyl salicylate related differentially expressed genes in NMA+ vs AMA+; Table S4: Methyl salicylate related differentially expressed genes in NMA− vs NMA+.
