# Supplementary material for: Arbuscular Mycorrhizal Fungus Alters Alfalfa (Medicago sativa) Defense Enzyme Activities and Volatile Organic Compound Contents in Response to Pea Aphid (Acyrthosiphon pisum) Infestation
Source: J Fungi (Basel). 2022 Dec 16;8(12):1308. doi: 10.3390/jof8121308 (PMC9787922; doi:10.3390/jof8121308)
Supplement: Supplementary file 1 [file jof-08-01308-s001.zip › Table S1.pdf]

Table S1. Main effects of AM fungus (inoculation or uninculation), pea aphid (infested or uninfested) and their two way interactions on AM colonization, Shoot fresh weight, Shoot dry weight, Shoot total N, Shoot total P, PPO, CAT, POD, SOD, SA, JA, ABA, NO, Trypsin inhibitor, Total phenols, Total VOCs. $P<0.05$ are highlighted in bold.

	**AM fungus**			**Aphid**			**AM fungus*Aphid**		
	DF	*F*	*P*	DF	*F*	*P*	DF	*F*	*P*
AM colonization	1	561.800	**0.0001**	1	0.200	0.6627	3	0.200	0.6627
Shoot fresh weight	1	12.344	**0.0043**	1	1.013	0.3341	3	0.012	0.9137
Shoot dry weight	1	8.034	**0.0151**	1	0.975	0.3429	3	0.015	0.9044
Shoot total N	1	9.941	**0.0083**	1	5.420	**0.0382**	3	0.316	0.5847
Shoot total P	1	6.431	**0.0261**	1	5.412	**0.0383**	3	5.479	**0.0373**
PPO	1	3.495	0.1063	1	114.903	**0.0001**	3	11.444	**0.0054**
CAT	1	2.692	0.1268	1	0.130	0.7252	3	6.020	**0.0304**
POD	1	0.150	0.7052	1	12.082	**0.0046**	3	0.923	0.3556
SOD	1	2.880	0.1154	1	0.102	0.7552	3	0.623	0.4452
SA	1	5.749	**0.0337**	1	0.548	0.4735	3	0.028	0.8696
JA	1	2.948	0.1116	1	5.758	**0.0335**	3	0.014	0.9086
ABA	1	0.204	0.6594	1	1.213	0.2923	3	0.204	0.6594
NO	1	2.111	0.1719	1	2.741	0.1237	3	0.917	0.3573
Trypsin inhibitor	1	7.596	**0.0174**	1	6.130	**0.0292**	3	0.369	0.5550
Total phenols	1	0.354	0.5629	1	0.001	0.9727	3	0.828	0.3807
Total VOCs	1	6.091	**0.0296**	1	2.196	0.1641	3	0.208	0.6569
