# Supplementary material for: Arbuscular Mycorrhizal Fungus Alters Alfalfa (Medicago sativa) Defense Enzyme Activities and Volatile Organic Compound Contents in Response to Pea Aphid (Acyrthosiphon pisum) Infestation
Source: J Fungi (Basel). 2022 Dec 16;8(12):1308. doi: 10.3390/jof8121308 (PMC9787922; doi:10.3390/jof8121308)
Supplement: Supplementary file 1 [file jof-08-01308-s001.zip › Table S2.pdf]

Table S2. The compounds of 114 VOCs in *Medicago sativa* roots inoculated with *Rhizophagus intraradices* (AM) and infested with *Acyrthosiphon pisum* (A+) or non-inoculated with *R. intraradices* (NM) and non-infested with *A. pisum* (A−).

ID	Class	Compounds
1	Terpenes	.gamma.-Terpinene
2	Terpenes	1−Cyclohexene−1−carboxaldehyde, 2,6,6−trimethyl−
3	Terpenes	1,3−Cyclohexadiene−1−carboxaldehyde, 2,6,6−trimethyl−
4	Terpenes	.beta.−Myrcene
5	Terpenes	.alpha.−Ionone
6	Terpenes	3−Buten−2−one, 4−(2,2,6−trimethyl−7−oxabicyclo[4.1.0]hept−1−yl)−
7	Alcohol	2−Penten−1−ol, (Z)−
8	Alcohol	1−Hexanol
9	Alcohol	1−Octen−3−ol
10	Alcohol	2−Octen−1−ol, (E)−
11	Alcohol	Benzyl alcohol
12	Aromatics	Phenol, 2−methoxy−
13	Alcohol	Phenylethyl Alcohol
14	Aromatics	Naphthalene
15	Heterocyclic compound	Pyridine
16	Aldehyde	2−Pentenal, (E)−
17	Aldehyde	Hexanal
18	Aldehyde	2−Hexenal, (E)−
19	Aldehyde	Benzaldehyde
20	Aldehyde	2,4−Heptadienal, (E,E)−
21	Aldehyde	Benzeneacetaldehyde
22	Aldehyde	2−Nonenal, (E)−
23	Aldehyde	2,6−Nonadienal, (E,Z)−
24	Aldehyde	2,4−Nonadienal, (E,E)−
25	Terpenes	2,6−Octadienal, 3,7−dimethyl−, (E)−
26	Aldehyde	Pentadecanal
27	Ketone	2−Heptanone
28	Ketone	3−Octanone
29	Ketone	3−Octen−2−one
30	Ketone	3,5−Octadien−2−one
31	Alkanes	Dodecane
32	Alkanes	Tridecane
33	Alkanes	Tetradecane
34	Alkanes	Pentadecane
35	Alkanes	Hexadecane
36	Alkanes	Heptadecane
37	Ester	Acetic acid, hexyl ester

38	Ester	Octanoic acid, methyl ester
39	Ester	Heptanoic acid, ethyl ester
40	Ester	Octanoic acid, ethyl ester
41	Ester	Methyl salicylate
42	Ester	Linalyl acetate
43	Aldehyde	2−octenal
44	Terpenes	(+)−alpha−Pinene
45	Ketone	4−Heptanone, 2,6−dimethyl−
46	Alcohol	Cyclohexanol
47	Aromatics	Benzene, 1,4−diethyl−
48	Ketone	5,9−Undecadien−2−one, 6,10−dimethyl−, (Z)−
49	Ester	2(4H)−Benzofuranone, 5,6,7,7a−tetrahydro−4,4,7a−trimethyl−, (R)−
50	Terpenes	3−Buten−2−one, 4−(2,6,6−trimethyl−1−cyclohexen−1−yl)−
51	Alkanes	Undecane, 4,6−dimethyl−
52	Alkanes	Dodecane, 4−methyl−
53	Alkanes	Decane, 2,3,5−trimethyl−
54	Alkanes	2,4−Dimethyldodecane
55	Alkanes	Dodecane, 4,6−dimethyl−
56	Alkanes	Tridecane, 2−methyl−
57	Alkanes	Pentadecane, 2,6,10−trimethyl−
58	Alkanes	2,6,10−Trimethyltridecane
59	Phenol	Phenol, 3,5−dimethyl−
60	Ketone	4−Hexen−3−one, 5−methyl−
61	Alkanes	Decane, 3−ethyl−3−methyl−
62	Alcohol	3−Methylpenta−1,3−diene−5−ol, (E)−
63	Aldehyde	Benzaldehyde, 3−ethyl−
64	Alkanes	Tetradecane, 4−methyl−
65	Aldehyde	Benzaldehyde, 2,4−dimethyl−
66	Alkanes	Hexadecane, 2,6,11,15−tetramethyl−
67	Heterocyclic compound	Furan, 2−hexyl−
68	Ester	2−Propenoic acid, 2−methyl−, (tetrahydro−2−furanyl)methyl ester
69	Ether	2−Propylphenol, n−propyl ether
70	Alcohol	10−Methyltricyclo[4.3.1.1(2,5)]undecan−10−ol
71	Aldehyde	2,5−Furandicarboxaldehyde
72	Alcohol	Cyclohexanol, 2,6−dimethyl−
73	Alkanes	Undecane, 6,6−dimethyl−
74	Olefin	Cyclohexene, 4−methyl−1−(1−methylethenyl)−
75	Alkanes	3,5−Dimethyldodecane
76	Alcohol	(E)−2,6−Dimethylocta−3,7−diene−2,6−diol
77	Alkanes	Undecane, 3,4−dimethyl−
78	Alkanes	Undecane, 3,8−dimethyl−

79	Alkanes	Nonane, 5−(2−methylpropyl)−
80	Alkanes	Dodecane, 5−methyl−
81	Alcohol	2−Ethyl−1−hexanol
82	Ester	Ethyl 4−(ethyloxy)−2−oxobut−3−enoate
83	Ketone	Cyclobutanone, 2,2,3−trimethyl−
84	Aromatics	Naphthalene, 2−methyl−
85	Heterocyclic compound	1H−1,2,4−Triazol−3−amine, 5−methyl−
86	Ester	Butanedioic acid, methyl−, dimethyl ester
87	Aldehyde	Benzaldehyde, 2,5−dimethyl−
88	Terpenes	Cyclohexanol, 1−methyl−4−(1−methylethylidene)−
89	Alcohol	6−Octen−1−ol, 3,7−dimethyl−, (R)−
90	Phenol	Phenol, 4−(1−methylpropyl)−
91	Heterocyclic compound	Precocene I
92	Ester	Phenol, 2−methoxy−4−(2−propenyl)−, acetate
93	Phenol	Phenol, 4−hexyl−
94	Ketone	2H−Pyran−2−one, 6−pentyl−
95	Ester	Acetic acid, 2−ethylhexyl ester
96	Aldehyde	4−Heptenal
97	Terpenes	(−)−delta.−Panasinsine
98	Ketone	1−Hepten−6−one, 2−methyl−
99	Terpenes	2,6−Dimethyl−2−trans−6−octadiene
100	Ketone	1−Decen−3−one
101	Terpenes	1, 1, 5−Trimethyl−1, 2−dihydronaphthalene
102	Aldehyde	Cyclohexanecarboxaldehyde
103	Ester	Formic acid, octyl ester
104	Ketone	1H−Pyrrole−2,5−dione, 3−ethenyl−4−methyl−
105	Ester	2−Butenoic acid, 2−methyl−, 2−methylpropyl ester, (E)−
106	Heterocyclic compound	Oxetane, 3−(1−methylethyl)−
107	Ketone	3,4−Hexanedione
108	Heterocyclic compound	Thieno[2,3−b]thiophene,2−methyl−
109	Ketone	2'−Ethoxyacetophenone
110	Ester	Octanoic acid, 3−hexenyl ester, (Z)−
111	Heterocyclic compound	Thiophene, 2−butyl−5−ethyl−
112	Ketone	5'−Hydroxy−2',3',4'−trimethylacetophenone
113	Aldehyde	2−Isopropyl−5−methylhex−2−enal
114	Ester	3−Hexen−1−ol, acetate, (E)−
