# Supplementary material for: Arbuscular Mycorrhizal Fungus Alters Alfalfa (Medicago sativa) Defense Enzyme Activities and Volatile Organic Compound Contents in Response to Pea Aphid (Acyrthosiphon pisum) Infestation
Source: J Fungi (Basel). 2022 Dec 16;8(12):1308. doi: 10.3390/jof8121308 (PMC9787922; doi:10.3390/jof8121308)
Supplement: Supplementary file 1 [file jof-08-01308-s001.zip › Table S3.pdf]

Table S3. Methyl salicylate related differentially expressed genes in NMA+ vs AMA+

Gene_ID	log2FC	P_{adjust}	regulated
MS.gene002309	2.640	0.01827	up
MS.gene002329	7.336	0.00729	up
MS.gene003889	7.806	1.9E-06	up
MS.gene006713	3.279	0.04206	up
MS.gene012415	8.548	0.00137	up
MS.gene032992	5.914	0.00429	up
MS.gene036189	7.624	0.0023	up
MS.gene055127	2.998	0.00453	up
MS.gene074385	2.924	0.03468	up
MS.gene23374	3.165	0.03496	up
MS.gene23894	11.221	5.7E-08	up
MS.gene33297	-4.315	0.0002	down
MS.gene34096	-7.795	0.03628	down
MS.gene43046	7.952	0.00562	up
MS.gene46289	6.637	0.04629	up
MS.gene47360	4.565	0.01492	up
MS.gene48828	-7.082	0.01211	down
MS.gene55264	7.325	0.01727	up
MS.gene75923	-7.648	0.00398	down
MS.gene81660	-6.795	0.01766	down
MS.gene81663	-6.795	0.01766	down
MS.gene81658	-6.795	0.01766	down
