# Supplementary material for: Arbuscular Mycorrhizal Fungus Alters Alfalfa (Medicago sativa) Defense Enzyme Activities and Volatile Organic Compound Contents in Response to Pea Aphid (Acyrthosiphon pisum) Infestation
Source: J Fungi (Basel). 2022 Dec 16;8(12):1308. doi: 10.3390/jof8121308 (PMC9787922; doi:10.3390/jof8121308)
Supplement: Supplementary file 1 [file jof-08-01308-s001.zip › Table S4.pdf]

Table S4. Methyl salicylate related differentially expressed genes in NMA− vs NMA+

Gene_ID	log2FC	Padjust	regulated
MS.gene003889	-7.193	0.02831	down
MS.gene012415	-8.877	0.00012	down
MS.gene23894	-9.313	0.04348	down
