# Supplementary figures and images for: Arbuscular Mycorrhizal Fungus Alters Alfalfa (Medicago sativa) Defense Enzyme Activities and Volatile Organic Compound Contents in Response to Pea Aphid (Acyrthosiphon pisum) Infestation
Source: J Fungi (Basel). 2022 Dec 16;8(12):1308. doi: 10.3390/jof8121308 (PMC9787922; doi:10.3390/jof8121308)

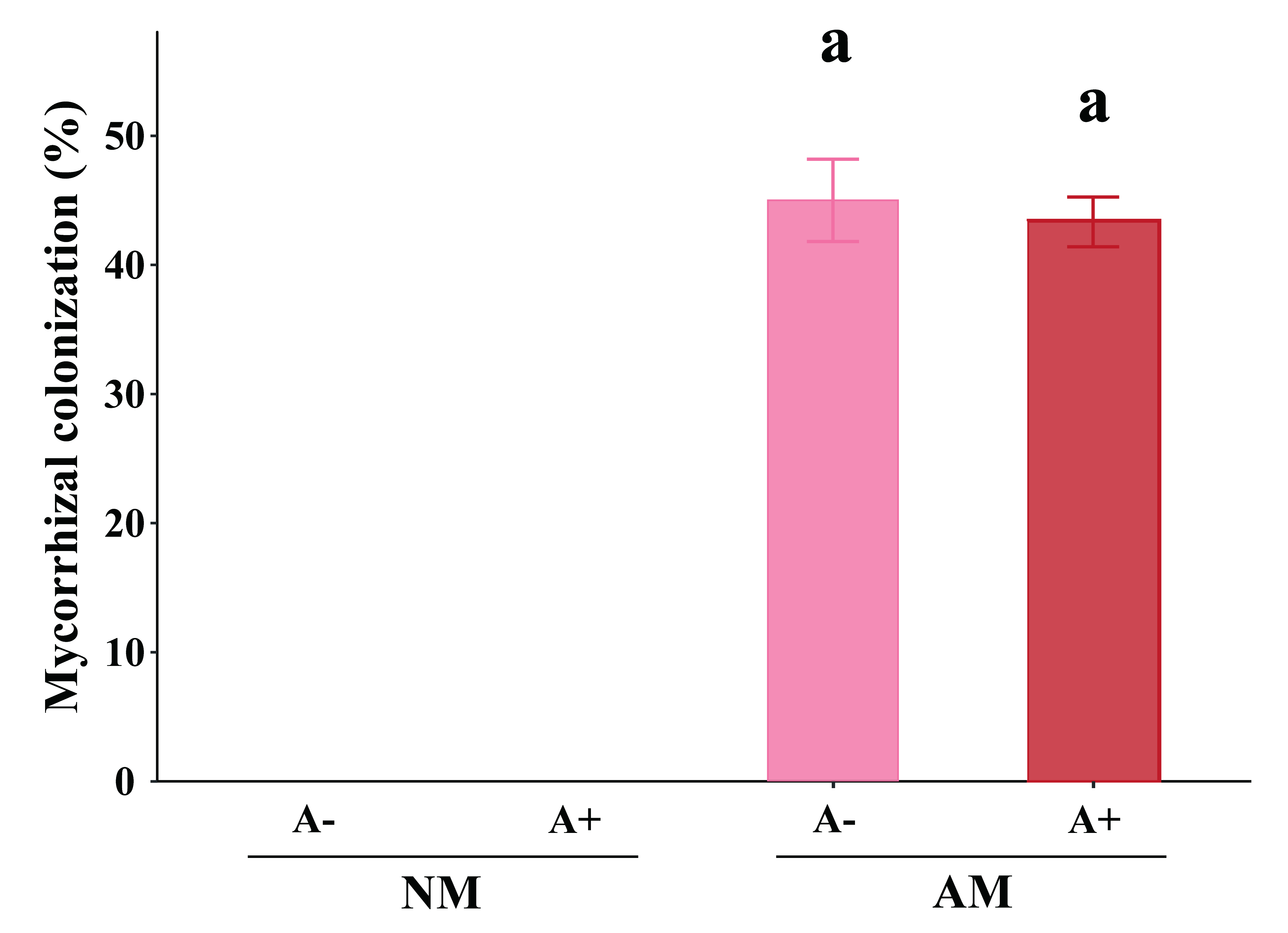

Supplement: Supplementary file 1 [file jof-08-01308-s001.zip › Figure S1.tif]

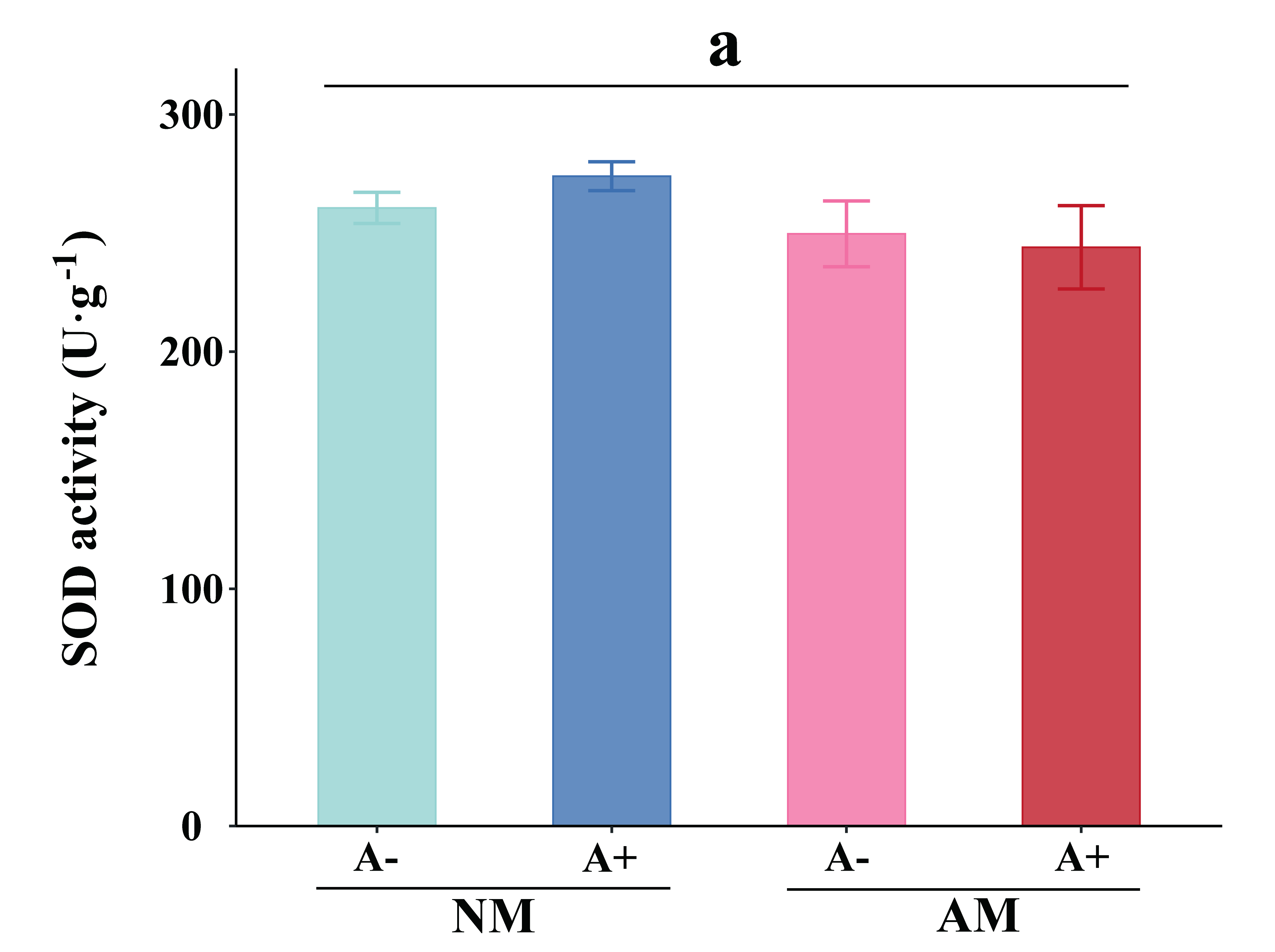

Supplement: Supplementary file 1 [file jof-08-01308-s001.zip › Figure S2.tif]

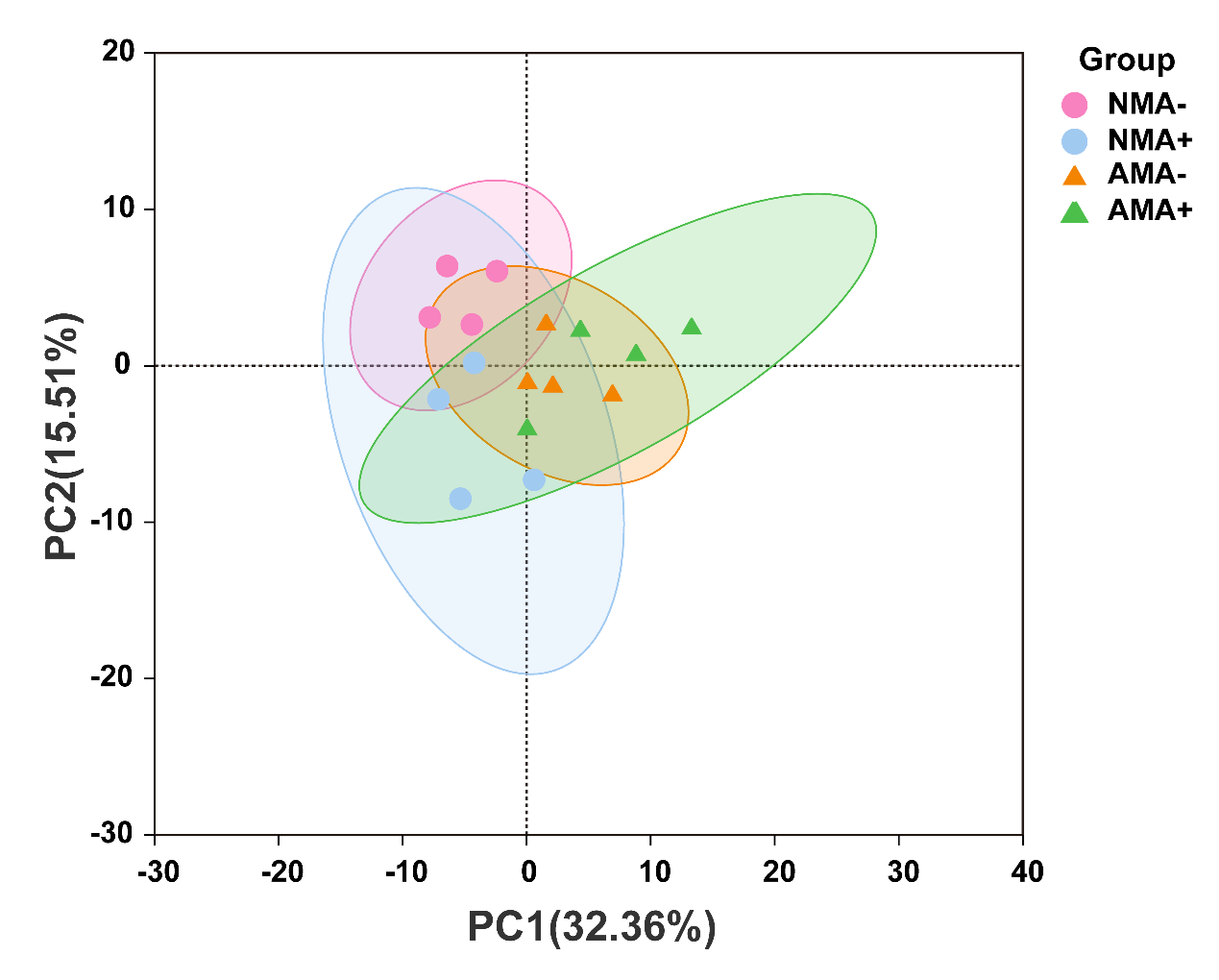

Supplement: Supplementary file 1 [file jof-08-01308-s001.zip › Figure S4.tif]
